# Supplementary material for: COVID-19 vaccine effectiveness among healthcare workers during the Omicron period in the country of Georgia, January – June 2022
Source: PLoS One. 2025 May 21;20(5):e0311337. doi: 10.1371/journal.pone.0311337 (PMC12094764; doi:10.1371/journal.pone.0311337)
Supplement: Attachment — Submitted filename: Response to Reviewers.docx [file pone.0311337.s002.docx]

**14 March 2025**

**Dear Plos One Editors,**

**Thank you and thanks to the reviewers for the thorough review of our manuscript. We have done our best to address all of the questions raised by reviewers in the point-by-point responses below.**

**Thanks again for considering our manuscript for publication.**

**Sincerely,**

**Caleb Ward**

1. **Please ensure that your manuscript meets PLOS ONE's style requirements, including those for file naming.**

We have changed the manuscript and file names to match PLOS ONE’s style requirements.

**2-3.  We note that the grant information you provided in the ‘Funding Information’ and ‘Financial Disclosure’ sections do not match.**

We have changed the funding information section to reflect the appropriate funders. The funders had no role in study design, data collection and analysis, decision to publish, or preparation of the manuscript.

**4. Data availability**

Data contain potentially identifiable and sensitive participant information and cannot be shared. The data availability statement has been updated.

**5. Ethics statement**

The ethics statement has been added to the methods section of the manuscript.

**6. Please include captions for your Supporting Information files at the end of your manuscript**

The following caption was added at the end of the manuscript.

Supporting Information. Ethics Approvals from NCDC and WHO.

**7. Please review your reference list to ensure that it is complete and correct.**

The reference list is complete and correct.

Reviewer #1: Abstract:

• Lines 12–15 (Abstract): The study presents original data on vaccine effectiveness (VE) among healthcare workers (HCWs) in Georgia during the Omicron period, a topic of significant public health interest. It appears that results have not been published elsewhere, but authors should explicitly confirm this in the manuscript.

We have added the line “but VE estimates against the Omicron variant have not been previously published” in the introduction.

Lines 1–37 (Abstract): The language is clear, but sentences such as “Absolute VE for a first booster was 40%… at ≥ 60 days” (Lines 28–30) could be simplified for readability.

We added “following vaccination” to the abstract to clarify the results so the sentence now reads:

Absolute VE for a first booster was 40% (95% Confidence Interval (CI) -56 – 77) at 7– 29 days following vaccination, -9% (95% CI -104 – 42) at 30 – 59 days following vaccination, and -46% (95% CI -156 – 17) at ≥ 60 days following vaccination.

Methodology and Analysis :
• Lines 61–116 (Methods): The cohort design and use of Cox proportional hazards models are appropriate. However:
• Provide more details on how inconsistent testing among HCWs (Line 73) was accounted for in VE estimation.

We did not account for variability in routine testing of asymptomatic participants. However, we focused our analysis on symptomatic infection, and outcome which is less likely to be affected by the variability in routine asymptomatic testing.

We have added the above text to the limitations section.

• Clarify why serology tests for inactivated vaccines could not determine prior infections (Line 97). Could this limitation bias results?

We could not use serology test results to determine prior infection for participants who were previously vaccinated with an inactivated vaccine (BBIBP-CorV or CoronaVac), as these vaccines routinely generate anti-nucleocapsid antibodies, and therefore positive serology test results in these participants could reflect either prior infection or prior vaccination.

We have added the additional language above to the methods section.

Because we could not use serology tests to determine previous infection status among the 22% of participants who had received inactivated vaccines, we likely underestimated the number of previous infections in this population. However, because we did not conduct stratified analysis based on previous infection, this underestimation should not have biased our VE estimates.

We have added the text above to the limitations section.

• Adjustments for confounders (Lines 106–109) are comprehensive, but their selection rationale should be better justified.

We have added the following sentence to the methods section: “These variables were chosen because they were previously found to be associated with vaccine uptake and effectiveness.”

• (lines:76-78)Serology samples were tested for anti-nucleocapsid antibodies and anti-spike antibodies by Roche Elecsys Anti-SARS-CoV-2 S immunoassay electrochemiluminescence immunoassay (ECLIA): what is the variant strain which was used as coated antigen in kits ? and what is the sensitivity of kit for detection the Ab against predominant circulating variants ?

Added to the limitations.

The ECLIA assay used antigens from the original SARS-CoV-2 strain.

We did not find any data on the sensitivity of these tests for antibodies from Omicron ba1 and ba2. We used antibody kits that detected the original strain and results may differ from the predominant strain in our study.

We could not find any published studies on PubMed or pre-print servers that evaluated the sensitivity of the ECLIA test against Omicron subvariants. However, one study that evaluated the sensititivity of ECLIA tests against the original strain found that the sensitivity of the test for IgG and IgM were 0.93 and 0.85.^1^

Because of this we added the following sentence to the limitations,

“We may have missed previous infections not captured by PCR testing or serology testing. While we could not find published data on the sensitivity of the ECLIA serology test against Omicron subvariants, the pooled sensitivity of the test against the original strain for IgG and IgM were 0.93 and 0.85, respectively, compared to rRT-PCR.”

^1^ ^Vengesai, A., Midzi, H., Kasambala, M.^ *^et al.^* ^A systematic and meta-analysis review on the diagnostic accuracy of antibodies in the serological diagnosis of COVID-19.^ *^Syst Rev^* **^10^**^, 155 (2021). https://doi.org/10.1186/s13643-021-01689-3^

• vaccine effectiveness (lines:80-99) why did not you use VNT to detect the vaccine antibody titer in vaccinated HCWs against predominant circulating variants?

We did not have the resources to conduct micro-neutralization assays to assess SARS-CoV-2 infection.

We have added this sentence to the limitations section.

Conclusions and Data Support :

• Lines 163–234 (Discussion):

• The conclusion of “moderate but waning VE” is supported by the data. However, the authors should elaborate on the implications of waning VE for booster policy in middle-income countries (Lines 231–234).

We added the following sentence to the conclusion:

The moderate but waning VE in this study against symptomatic and medically attended infection, combined with the more durable COVID-19 VE against severe outcomes described in other studies, underscores the need for broader efforts in Georgia to increase vaccine coverage among priority groups such as HCWs, and to encourage the adoption of guidelines that encourage high-risk individuals to receive annual COVID-19 revaccination, as recommended by WHO.

• Lines 163–234 :The writing is coherent, but the overuse of technical terms may limit accessibility to a broader audience. Simplify where possible.

We have attempted to simplify the language and limit the use of technical terms where we could.

• Discuss whether high prior infection rates (Line 122) could have influenced observed VE.

We added the following sentence to the limitations, “The high rates of prior infections among participants likely led to an underlying level of immunity not seen in uninfected populations. While the immunity likely blunted the magnitude of VE, it reflects the real world setting of Georgia at the time of the study.”

General Recommendations:

1. Figures and Tables: The figures (e.g., VE over time) are informative but need better integration into the discussion to illustrate key findings.

We have integrated the tables and figures more into the discussion and referenced them in the text where relevant.

2. Limitations (Lines 221–227): Expand on the potential selection bias due to voluntary participation and address whether the sample is representative of HCWs in Georgia.

We added the following to the limitations, “Participants may have been healthier and more likely to be compliant with the study criteria compared to HCWs in the hospital. However, we do not know if this would have an overall impact on VE. While the hospitals included in our study are large hospitals in the Georgian capital, we do not have demographic and comorbidity data for all Georgian HCWs, and therefore we are unable to determine the extent to which our study population is representative of all HCWs in the country.”

3. Implications for Future Research: Suggest areas for further study, such as VE against severe outcomes.

Reviewer #2: he study report on COVID-19 vaccine efficacy (VE) against symptomatic infection from January 2022 to June 2022, during the Omicron era in Georgia. Participants were selected from healthcare workers vaccinated with either mRNA or inactivated vaccines available. The report indicates that the booster dose is moderately effective in preventing symptomatic infection in healthcare workers (HCWs) in Georgia, but its effectiveness wanes over time. These findings have been previously reported in other countries with similar conclusions, and the same authors had published a similar study (https://doi.org/10.1371/journal.pone.0307805). In this study, the authors determined the VE of COVID-19 vaccines containing the ancestral virus during Omicron circulation. The authors should discuss other studies that reported the VE of ancestral monovalent vaccines against Omicron. Furthermore, some participants received inactivated vaccines, while others received mRNA vaccines. The results should be interpreted cautiously since both vaccines are produced differently. Therefore, it is not clear whether these findings and conclusions represent the VE of mRNA or inactivated vaccines. Authors should comment and discuss on this in the discussion.

We added the following sentence to the discussion, “Our finding of moderate VE against symptomatic infection that rapidly waned during Omicron is similar to findings from other studies showing moderate VE using ancestral monovalent vaccines against Omicron that decreased over time.” ^1,2^

1. Andrews N, Stowe J, Kirsebom F, Toffa S, Rickeard T, Gallagher E, et al. Covid-19 Vaccine Effectiveness against the Omicron (B.1.1.529) Variant. New England Journal of Medicine. 2022 Apr 21;386(16):1532–46.
2. Kirsebom FCM, Andrews N, Stowe J, Toffa S, Sachdeva R, Gallagher E, et al. COVID-19 vaccine effectiveness against the omicron (BA.2) variant in England. The Lancet Infectious Diseases. 2022 Jul 1;22(7):931–3.

We added the following sentence to the discussion, “Our overall VE results should be interpreted cautiously as we used both inactivated and mRNA vaccines in our study.”

Minor edit: The Sinopharm vaccine is written as BBIBP-CorV in some places, but used differently as BIBP-CorV in other places. Please correct.

We corrected BIBP-CorV to BBIRP-CorV and the term is consistent throughout the manuscript.
